# A rare case of urinary tract infection due to Trichosporonasahii in a diabetic patient

**DOI:** 10.11604/pamj.2015.20.127.6152

**Published:** 2015-02-13

**Authors:** Maryem Iken, Ahmed Belkouch, Badia Bellarj, Hafida Naoui, Laila Boumhil, Anass El Bouti, Said Jidane, Lahcen Belyamani, Badreddine Lmimouni

**Affiliations:** 1 Parasitology and Mycology Department, Mohamed V Military Hospital of Instruction, Rabat, Morocco; 2 Emergency Department, Mohamed V Military Hospital of Instruction, Rabat, Morocco

**Keywords:** urinary tract infection, Trichosporon asahii, amphotericin B

## Abstract

Trichosporonasahii is a basidiomycete yeast responsible for white piedra and onychomycosis in the immunocompetent host. In the immunocompromised patients, invasive infections are reported; their diagnosis is difficult and they are associated with high mortality rate. Urinary infection due to Trichosporon Asahi is rare but its incidenceis increasing. We report the case of a 58 year old diabetic patient. The yeast was isolated from urine samples of three consecutive crops in pure form. The patient improved after antifungal therapy.

## Introduction

Yeasts of the genus Trichosporonsp are cosmopolitan, widespread in the environment (soil, water, organic substrates, plants). They are also part of the occasional saprophytic flora in humans at the skin, mucous membranes, and gastrointestinal tract [1, 2]. Six species are considered potentially pathogenic for humans: T. asahii, T.cutaneum, T.inkin, T.asteroides, T.mucoides, T.ovoides [2–5]. These basidiomycete yeasts can be, depending on the species, responsible for benign superficial infections as white piedra of the hair in the different locations of the body, deep localized or disseminated infections especially among vulnerable patients [2, 4, 5]. Rare cases of systemic infections were attributed to other species such T.loubieri and T.pullulans [6]. Several cases of hypersensitivity pneumonitis T. asahii were reported exclusively in Japan in patients with high titer antibodies [7]. We report the case of a urinary tract infection with T. asahii diagnosed in the parasitology and Mycology department of the Mohamed V Military Hospital of instruction in Rabat.

## Patient and observation

A 58 years old, diabetic patient treated by insulin since 10 years, he has undergone an amputation of his left foot a year ago due to gangrene. He was admitted to the emergency department for the management of a febrile urinary tract infection. Laboratory tests on admission revealed acute renal failure (urea 1,2 g/l, creatinine 68 mg/l) and C-reactive Protein was at 300 mg/l). The blood count showed leukocytosis of 17,000 per mm3, normochromic normocytic anemia with hemoglobin at 7.1 g/dl. The patient was given antibiotics (Ceftriaxone). An initial urine culture came back positive for E. coli sensitive to Ceftriaxone. A significant clinical improvement was noted but a persistent inflammatory syndrome has led us to realize another infectious analysis. Thus, three cytobacteriological urine analysis made on day 15, 17 and 20 of his hospitalization were positive. The macroscopic examination objectified colonies of white creamy yeast, rough, dry and cerebriform, with irregular contours. Microscopic examination, showed voluminous elongated yeast with arthrospores or arthroconidia, hyphae were also observed, typical of the genus Trichosporon [4, 5, 8, 9]. The isolation of the same yeast on three consecutive urine cultures and the absence of any bacterial outbreak, has allowed us to consider T.asahii as the etiologic agent of urinary infection. The species identification was based on the study of the biochemical characteristics by Auxacolor TM 2 gallery (BIO-RAD, France) and was verified by the VITEK 2 Compact YST cards system (bioMérieux, France). The study of antifungal susceptibility on the VITEK 2 showed that the strain is susceptible to amphotericin B, fluconazole, to 5-flucytosine and voriconazole. Samples from the mouth after the occurrence of oropharyngeal candidiasis isolated Trichosporonasahii. The patient was given amphotericin B. He completely improved.

## Discussion

The Trichosporonsp was described as an emerging opportunistic agent. Indeed, this basidiomycete yeast is increasingly involved in deep infections. It is the second cause of invasive infections in patients with haematologic malignancies profoundly neutropenic after Candida yeast species [10, 11]. In the various series reported in the literature, acute leukemia is the most frequent pathology found in 82% of cases of disseminated trichosporonosis [12]. The increased incidence of trichosporonosis is associated with other predisposing factors as immunosuppressive or corticosteroids therapy especially, a human immunodeficiency syndrome, organ transplantation or extensive burns [5, 6, 9, 11, 13]. 16 (84%) of the 19 cases of trichosporonosis described by Ruan et al, were due to Trichosporonasahii [11]. This species is recently considered as one of the causative agents of deep localized trichosporonosis. Urinary infection by T.asahii is rarely reported in the literature [9, 14], but its incidence has increased in the recent years in hospital due to many factors including prolonged intravenous and bladder catheterization, cardiac prosthetic valves, peritoneal dialysis and broad-spectrum antibiotics [3, 13, 15]. The recent review of Sun Wei & al shows that of 23 patients with urinary tract infection with T. asahii, 14 (60.8%) had urinary catheterization [12]. Various morbidity factors were reported in the same study: hypertension, heart failure, chronic obstructive pulmonary disease, hemodialysis. Diabetes is noted in 34.7% of cases (8/23). AgenorMessias& al reported a high prevalence of T.asahii 76.66% (n = 23) isolates from urine samples and catheters, followed by T. Inkin and T. asteroides which represent a prevalence of 16.66% (n = 5) and 6.66% (n = 2) of isolates [16]. The same study describes the potential colonization of the region by perigenital six species of Trichosporonspp in normal immunocompetent population (T.asahii, T. asteroides, T. ovoid, T. mucoid, T.inkin, T. cutaneum) a rate of 11.15%. This rate confirms the one recorded by Pini G & al (12.4%) in their study [17]. Therefore, in our case, the Yeasts would probably colonized the bladder catheter from the perigenital skin flora during catheterization [3, 16, 18]. In addition to their usual skin saprophytism the Trichosporon spp. have the ability to colonize other sites [5]. Virulence may be related to host defense. Indeed, the profound trichosporonosis occur in immunocompromised patients with impaired cellular immunity and macrophage functions. This virulence may also depend on the species itself. T. asahii secretes β-N-acetylhexosaminidase, the enzyme responsible for the degradation of the oligosaccharides present on the surface of macrophages and preventing recognize the fungal cells [19]. Recently, Wei Sun & al have detected two virulence factors of T. asahii: haemolytic activity and the biofilm formation capacity on polystyrene surface. These two factors give this yeast pathogenicity in urinary tract infections to Trichosporonasahii [12]. The optimal antifungal treatment of deep trichosporonosis remains controversial despite the apparent effectiveness of some antifungal in vitro [5, 20]. Trichosporonspp are inconstantly sensitive to amphotericin B, the reference antifungal, and to 5-fluorocytosine [5, 9, 20]. Several authors report their studies of the minimum inhibitory concentration (MIC)of amphotericin B which is often elevated about 0.25 to 4mg/ml [21]. On the other hand, Wei Sun & al used the technique recommended by the Clinical and Laboratory Standards Institute using the method CLSI M27-A3 [22], to test in vitro the sensitivity of 23 strains of T. asahii to amphotericin B, 5-flucytosine and fluconazole, itraconazole, voriconazole. Their results determined low MICs for amphotericin B (0.5 mg/ml). Furthermore, voriconazole has in vitro activity higher than that of amphotericin B and fluconazole on the isolates of T. asahii [12, 23].

## Conclusion

Trichosporonasahii is an emerging yeast responsible of deep localized infections reported mainly in the immunocompromised patients. Although rare, they are difficult to diagnose and have a guarded prognosis. The mycological study based on direct examination allows to set up the diagnosis and start rapid and effective treatment.
